# Pulmonary actinomycosis mimicks lung cancer

**DOI:** 10.1590/0037-8682-0195-2022

**Published:** 2022-08-12

**Authors:** Yener Aydin, Remzi Arslan, Mustafa Filik

**Affiliations:** 1 Ataturk University, Medical Faculty, Department of Thoracic Surgery, Erzurum, Turkey.; 2 Ataturk University, Medical Faculty, Department of Pathology, Erzurum, Turkey.; 3 Ataturk University, Medical Faculty, Department of Nuclear Medicine, Erzurum, Turkey.

**FIGURE 1:**
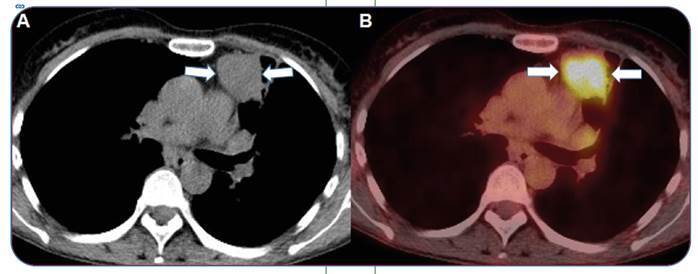
F-18 FDG PET/CT scan shows a lesion approximately 53 mm in size (arrow) with irregular borders in the anterior segment of the **(A)** left lung upper lobe and **(B)** an increased maximum standardized uptake value of 16.7. F-18 FDG PET/CT, 2-deoxy-2-[fluorine-18] fluoro-D-glucose positron emission tomography/computed tomography.

A 54-year-old woman presented with complaints of cough, fever, sputum, chest pain, and hemoptysis. Lung cancer was considered after viewing the patient’s positron emission tomography/computed tomography scan (Figure 1). Furthermore, a diagnosis could not be made based on the bronchoscopy and tru-cut biopsy results. Since the patient’s complaints of hemoptysis gradually increased, we performed a left upper lobectomy. Pulmonary actinomycosis was diagnosed as a result of the histopathological evaluation. 

Pulmonary actinomycosis is a very rare disease that usually occurs in people with poor oral hygiene and can cause serious morbidity and mortality if not treated appropriately. The infection is caused by a form of *Actinomyces*, an anaerobic bacterium with a progressive course. Pulmonary actinomycosis is difficult to diagnose. Clinically and radiologically, pulmonary actinomycosis can often mimic tuberculosis, lung abscess, or lung cancer^1^ ^,^ ^2^, and can sometimes cause life-threatening recurrent hemoptysis^3^. Pulmonary actinomycosis is a rare condition that should be considered in the differential diagnosis of lung cancer.
